# Developing the hydrological dependency structure between streamgage and reservoir networks

**DOI:** 10.1038/s41597-020-00660-6

**Published:** 2020-10-01

**Authors:** Sudarshana Mukhopadhyay, A. Sankarasubramanian, Chandramauli Awasthi

**Affiliations:** 1 Department of Biological and Environmental Engineering, Cornell University, Ithaca, NY USA; 2 Department of Civil, Construction and Environmental Engineering, North Carolina State University, Raleigh, NC USA

**Keywords:** Hydrology, Ecology, Environmental sciences

## Abstract

Reliable operation of physical infrastructures such as reservoirs, dikes, nuclear power plants positioned along a river network depends on monitoring riverine conditions and infrastructure interdependency with the river network, especially during hydrologic extremes. Developing this cascading interdependency between the riverine conditions and infrastructures for a large watershed is challenging, as conventional tools (e.g., watershed delineation) do not provide the relative topographic information on infrastructures along the river network. Here, we present a generic geo-processing tool that systematically combines three geospatial layers: topographic information from the National Hydrographic Dataset (NHD*Plus*V2), streamgages from the USGS National Water Information System, and reservoirs from the National Inventory of Dams, to develop the interdependency between reservoirs and streamgages along the river network for upper and lower Colorado River Basin (CRB) resulting in River and Infrastructure Connectivity Network (RICON) that shows the said interdependency as a concise edge list for the CRB. Another contribution of this study is an algorithm for developing the cascading interdependency between infrastructure and riverine networks to support their management and operation.

## Abstract (summary)

**Table:** 

Measurement(s)	network connectivity of streamgages and reservoirs
Technology Type(s)	geospatial analysis • computational modeling technique • digital curation
Factor Type(s)	location of USGS streamgages • location of NID reservoirs
Sample Characteristic - Environment	drainage basin • reservoir
Sample Characteristic - Location	Colorado River

Machine-accessible metadata file describing the reported data: 10.6084/m9.figshare.12757940

## Background & Summary

**Fig. 1:**
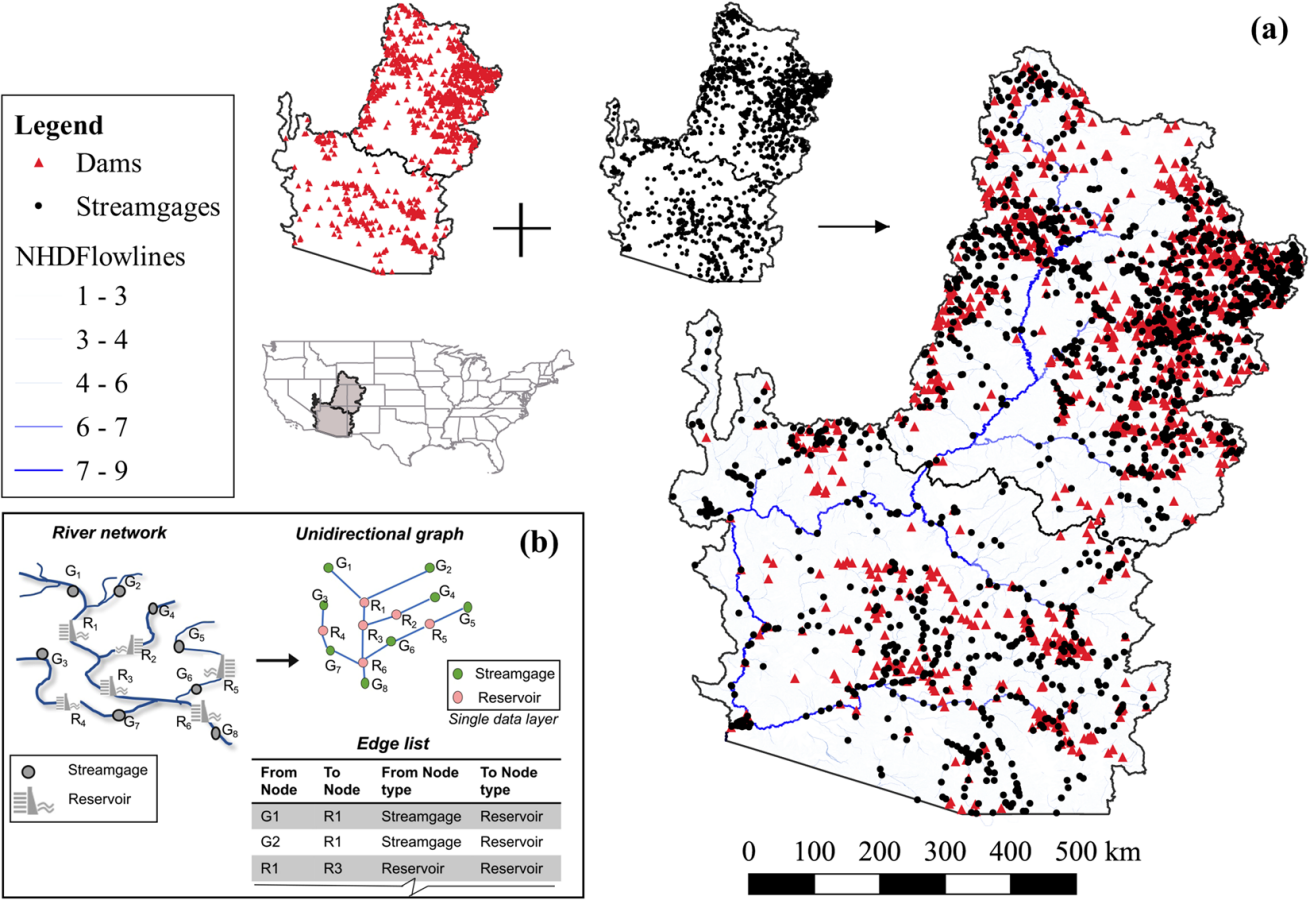
(**a**) Study area: Dams (red triangles) and streamgages (black circles) locations in the Colorado River Basin. *NHDFlowlines* are shown in gradation of blue – lighter color indicates lower Strahler stream order. Figure developed using QGIS^32^ (**b**) Basic idea of the proposed approach. In the unidirectional graph, edges (blue lines) contain all attributes of NHD*Plus*V2 flow lines that connect pairs of nodes (green and orange circles).

Understanding the vulnerability of critical infrastructure to natural disasters such as riverine and coastal floods requires hydroclimatic information on the magnitude and frequency of extreme events and their impact on flood control structures (e.g., reservoirs, levees, and dikes))^1,2^. The predicted magnitude of hydrologic extremes should be linked with the critical information for emergency response: how far they are from rivers or drainage networks and disaster source^3^. For instance, critical infrastructure such as nuclear power plants require proximity to rivers for cooling the reactors, but such proximity also increases the flood risk. Similarly, for the regular operation of thermal power plants, it is essential to identify safe locations for water withdrawal and discharge the dissolved-oxygen-depleted water back in the river. Similar arguments could be extended to the operation and management of other infrastructures (e.g., wastewater treatment plants, reservoirs, bridges) and their dependency on the rive network for monitoring riverine conditions. It is often needed to coordinate the operation of various infrastructures based on river conditions (e.g., coordinating upstream power-plants discharges based on flow conditions). Hence the dependency of critical infrastructures (e.g., reservoirs, power plants) across the riverine network must be known as a priori for operation and management. Developing this interdependency information requires geospatial referencing of the critical infrastructure with the riverine network. Although most agencies have manually developed this cascading interdependency to support the regular operation, developing this structure upfront over a large spatial scale is still challenging using simple watershed delineation or other geospatial processing tools. Despite the advances in representing river network with the detailed attributes in high-resolution National Hydrography Dataset^4,5^ (NHD*Plus*V2), there are no tools or software packages available to geospatially represent the infrastructure dependency with streamgages and other entities that rely on the entire river network (Fig. 1). Current approaches towards referencing infrastructure dependency with streamgage networks and other infrastructures are purely place-based and developed manually without a generic tool to develop the interdependency structure over a large spatial scale. To our knowledge, information on riverine connectivity between reservoir and stream gauges is identified locally, and such information lies with agencies responsible for the operation.

No systematic database exists for large river basins that provide the riverine connectivity between reservoirs and stream gauges. Using detailed river network information in NHDP*lus*V2, we develop an algorithm that develops the riverine connectivity between reservoirs and stream gauge for one of the highly dammed river basins, the upper and lower Colorado River basins, from the western US.

This study’s primary intent is to develop a geospatial tool that develops the interdependency of reservoir network and streamgage network based on the river network described by NHD*Plus*V2^4,5^. NHD*Plus*V2 is a set of geospatial data products that is built based on high resolution (1:10000-scale or better) National Hydrography Dataset (NHD) and 10-meter National Elevation Dataset (NED). This database was first released in 2006 as its first version (NHDPlusV1). Later, it was enhanced by using more detailed input datasets, improved networking, and more accurate and consistent ‘Value Added Attributes’ (VAA’s) released as NHDP*lus*V2 in 2011. Digital stream network, NHDP*lus*V2, provides rich information on river connectivity representing the surface water of the United States^4,5^. NHDP*lus*V2 also includes detailed stream-catchment data^6^ (Stream-Cat) and lake-catchment data^7^ (Lake-Cat). Both Stream-Cat and Lake-Cat databases have rich information with (a) delineated basins (b) basins connectivity, (c) lake connectivity with upstream basins, and (d) basin or lake characteristics (e.g., land use data, soils, climate, etc.)^8–11^. Studies have used a parallel river routing framework for developing runoff data for the Mississippi River Basin using NHDP*lus*V2 dataset^5,12^. Recently, *Hydrolinks*^13^*–* an *R*^14^ package – was developed to demonstrate the usefulness of this high-resolution dataset in linking water quality monitoring gages to streams and lakes^13^. However, it did not identify the upstream-to-downstream dependency. For river basin management, we are often interested in relating the stream or lake network connectivity with other networks (e.g., power plants, reservoirs) that rely on rivers for their functionality. Further, reservoir networks often require information on upstream and downstream streamgages for effective monitoring of inflows and releases from the cascade. Even though individual reservoir systems have information on which gages should be monitored for operation or management, developing such information at the river basin or regional scale, which is often available as a policy document or reservoir operation manual, is a daunting task. Further, small and medium-size reservoirs (height smaller than 100 feet or storage less than 50000 Acre-Feet)^15^ are mostly owned by private agencies, and such dependency information are not readily available. As spatially distributed point data have complex geographic connectivity on the ground, it poses a significant challenge for geo-statistical analyses. For example, a hydrologic network is regulated by streamflow channel connectivity as opposed to their geographic proximity^16^. Thus, Euclidean distance-based methods cannot address the hydrological connectivity, the relative position of sites in a river system based on the river connectivity is often needed. Given reservoir networks and streamgage networks are connected by the stream network, we propose an algorithm that effectively provides the upstream and downstream connectivity of a given reservoir with streamgage network and the other reservoirs in the basin.

This study aims to develop an algorithm for identifying the interdependent structure between the streamgage network and reservoir network for large river basins using the comprehensive river network attributes available from NHD*Plus*V2. Effectively, the presented algorithm in *R*^14^ Reservoirs-Infrastructure Cascade (RIC), develops a tree data structure that provides the parent-child relationship between reservoirs and stream gages over the entire river basin as RICON database^17^. The developed algorithm is applied and demonstrated for the upper and lower CRB. The application of the algorithm produced a tree data structure that consists of reservoir-streamflow interdependency based on 1344 dams and 1656 streamflow gages from water resources regions 14 and 15 over the CRB (Fig. 1). The manuscript is organized as follows: The next section describes the methodology, including data preparation and the algorithm, followed by the discussion of the developed Reservoir-streamgage network database RICON^17^. Finally, we discuss the validation of the database and its availability and data retrieval.

## Methods

Developing the interdependency between reservoirs and streamgages across a river network (Fig. 1a) is essentially merging three data sets (attributes of dams, streamgages, and river flow lines) (Table 1) to create a unidirectional graph or network^18,19^. In the resulting graph, each node of the network represents a streamgage or reservoir with the connecting stems between pairs of nodes represent river reaches in-between those streamgages or reservoir pairs (Fig. 1b). In this merged unidirectional graph, point information can be stored as attributes as the node of the unidirectional graph. We represent a reservoir-streamgage network as an ‘edge list’^18,19^ a table of information defined by flow connectivity between network nodes (streamgage or reservoir). In this edge list, each row is a connection link (edge) from one node to another containing *NHDPlusflowlines*^4^ attribute such as unique identifiers (*ComID*) and distances along the river path.

**Table 1:** Data sources.

Name	Data type	# of features	Source	Data file
National Inventory of Dams (NID)	Reservoir information	1344	Retrieved using *R* package *dams*	nid_df.csv
NWIS gages	Streamgages	1656	Accessed using *R* package *DataRetrieval*	sites_df.csv
NHDP*lus*V2	Flow line features and value-added attributes		http://www.horizon-systems.com/NHDPlus/NHDPlusV2_data.php	NHD_data.RData (combined NHD*Plus*V2 input used for regions 14 and 15)

We provide our dataset as an edge list that can be easily adopted in various geospatial applications. The motivation to present the combined dataset in a robust format is to enable end-users from varied backgrounds to use the dataset in a conventional network data format. For clarity, hereafter, we refer to the nodes of our unified edge list as ‘points’ which can be either a dam or a streamgage. In the rest of the article, attributes of NHD*Plus*V2 are summarized in Table 2 and written in italics. We use the word ‘node’ only in the context of *NHDFlowlines*’ connecting nodes and its attributes.

**Table 2:** Glossary of terms.

PlusFlowlinesVAA	Value Added Attributes (VAAs) for each NHDFlowline feature.
Plusflow	A table consisting of flowing and non-flowing connections between NHDFlowline features.
ComID	Common identifier of an NHDFlowline feature
HydroSeq	Hydrologic Sequence Number of NHDflowline features ordering them from upstream to downstream
DnLevelPat	Downstream mainstem level path identifier
ArbolateSum	Distance of the downstream end of each NHDFlowline feature from the headwaters (in kilometres)
LevelPathID	Leval Path Identifier – numbered in a way that all segments of a given river (for example Colorado River) have the same identifier in the entire dataset.
TerminalFl	Indicator denoting if a NHDflowline feature is a terminal segment (1) or not (0)
ToNode	Unique identifier of the end point of a NHDFlowline feature
FromNode	Unique identifier of the starting point of a NHDFlowline feature
StartFlag	Indicator denoting if a NHDflowline feature is a headwater segment (1) or not (0)
LengthKM	Length of each NHDFlowline feature (kilometres)
ToNodeDirection	Direction of ToNode – end point of a NHDFlowline feature. Values of ToNodeDirection can be one of (a) 714 – coastal connection, (b) 709 – flowing connection, (c) 712 – headwater and (d) 713 – network end.

**Fig. 2:**
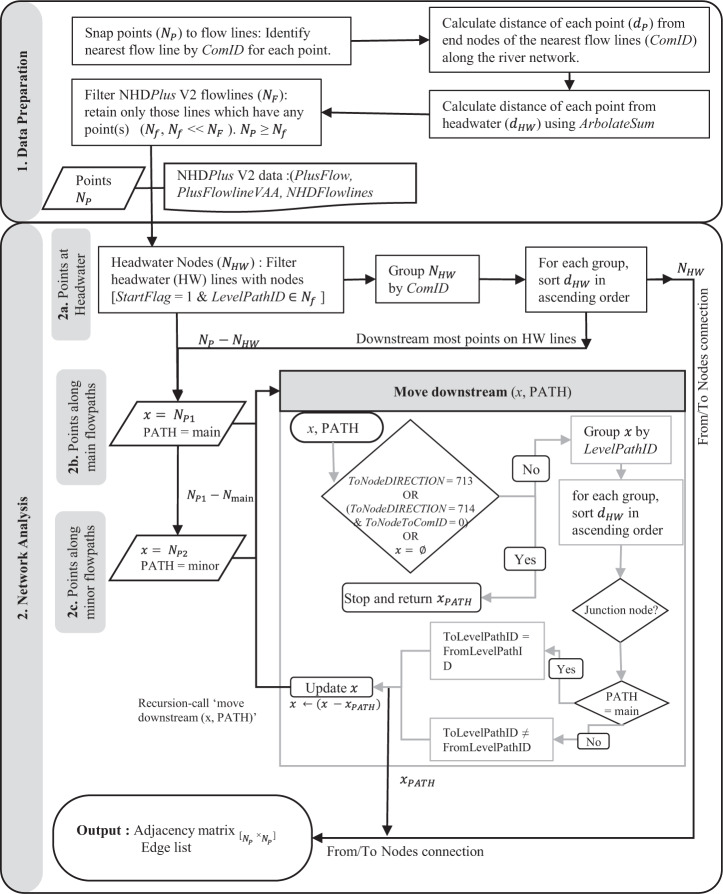
Schematic of the methodology. Words in italics font indicate attributes from the NHD*Plus*V2 database.

Combined reservoir-streamgage data is created by traversing on the *NHDPlusflowlines*^4^ network from headwaters to the downstream-most node(s) of each watershed, in a parallel fashion. The proposed method is summarized schematically in Fig. 2. We used various C++ ^20^ based *R* packages (*dplyr*^21^ and *sf* ^22^) for data handling and geospatial analysis. Headwater flow lines that have at least one point are analyzed first. Points (reservoirs or streamgages) are ordered according to their increasing distance from headwater based on ascending values of *ArbolateSum* of each *NHDFlowline* feature. At first, for each point, its nearest *NHDFlowline* and its *ComID* are recorded. Thus, each river reach where any streamgage or reservoir is located, all vector attributes of that flow line can be accessed from NHD*Plus*V2^4^ database. Nearest *NHDflowlines* of these points are grouped by *LevelPathID*, which ensures all features along a river are uniquely grouped. Next, a parallel node search along the downstream direction is carried out for each group identified in the previous step based on *LevelPathID*. The downstream parallel node search is essentially a depth-first-search^18,19^ – a network traversal technique that recursively explores downstream point features (reservoirs or streamgages) till all points along a river (grouped by *LevelPathID*) are identified. At this iterative step, we account for different conditions such as river junctions (e.g., divergences, convergences, or both), boundary of a watershed, isolated network, and coastlines or end of a stream reach.

For the application of the proposed algorithm to CRB, we relate spatial information (latitude, longitude) of each point (reservoirs or streamgages) along with attributes such as (1) unique identifiers (NID ID for reservoirs and USGS site number for streamgages), (2) *ComID* of nearest *NHDFlowline*, (3) distance of each point from end nodes of the nearest *NHDFlowline*, and (4) distance between each point and its immediate upstream and downstream point. Additional information can also be retrieved from the output edge list for specific applications depending on specific requirements of a network analysis problem. For example, to understand the cumulative effect of dams in CRB on flow alteration in the downstream river reach, a subset of network is used from the edge list where network nodes are the reservoirs and streamflow at gages located immediately downstream of these dams are the variables of interest^23^. Thus, the developed riverine connectivity between streamgage and reservoirs can be used to extract additional information (e.g., reservoir storage, drainage area of the streamgage, etc.) from the individual nodes.

### Input data sources

For CRB, the dataset combines dams^24^ from the National Inventory of Dams (NID) and streamgages^25^ from the United States Geological Survey (USGS) (Fig. 1a, Table 1). The NID^24^ is a database from the US Army Corps of Engineers (USACE) documenting information about more than 90,000 water control infrastructures (mainly dams) across the CONUS and US territories. This dataset includes information about a dam’s type, purpose, size, location, and classification of downstream potential hazard. According to this dataset, there are 1344 dams within the CRB (Water Resources Regions 14 and 15), which we used for the present study. Information on dams was retrieved using the R package *dams*^26^.

The National Water Information System (NWIS) database contains point measurements of water-related data (e.g., surface water, groundwater) collected at more than 1.5 million sites across the CONUS. This dataset contains records from 1899 to present water data for the nation. Around 20,000 streamflow gage stations are included in the NWIS dataset, of which 1656 are located within CRB (Regions 14 and 15).

In order to characterize the CRB stream network, we used high-resolution NHD*Plus*V2 dataset^4^ available from ftp://ftp.horizon-systems.com/NHDplus/NHDPlusV21/Data/. Our main purpose in utilizing the NHD*Plus*V2 database is to obtain high-quality streamflow lines and attributes (characterized by VAA), acting as the basis of upstream and downstream identification of dam-streamgage interdependency. We consider vector data of all input spatial datasets to develop the interdependent structure between reservoirs and streamgages over the CRB. For developing the RICON^17^ database using Vector Processing Units (*VPU’s*) in NHD*Plus*V2 dataset for watershed regions 14 and 15, the network analysis is carried out over the entire upper and lower CRB to maintain continuity in the river network from Glenn Canyon dam (outlet of water resources region 14) to Hoover dam which is located approximately 590 kilometers downstream in region 15.

### Data preparation

In geospatial data merging, if two or more-point shapefiles are used, one can simply join the attribute tables of each shapefile by a common ID. Using unique identifiers of each of the data sets, we can concatenate any string pattern (e.g., “Point.”, “Pt”) to make a simple, unique identifier (node number) for the merged points. Given the number of points or nodes *NP* (*NP* = *NR* + *NG*) where *NR* is the number of reservoirs and *NG* is the number of streamgages. At this stage, we only keep common IDs such as latitude, longitude, and point name and river information, if available, for the merged point dataset. For network analysis, we do not retrieve any other attributes to reduce the memory requirement. In data preparation, the first step is to identify the nearest river reach of each point feature (reservoirs or gages). This can be achieved easily using any available geographic information tool. We used the nearest feature identification technique in *sf* package^22^ in R along with string matching for identifying the nearest NHDflowline feature. This step can be computationally demanding, depending on the size of the datasets and geospatial tools used. Accuracy of this step is also subjected to the resolution of the river network data.

For each node (reservoir or streamgage), (1) nearest NHDflowline and (2) distance from either endpoint of the nearest flowline to the node must be recorded in this step (see data preparation panel in Fig. 1). The nearest NHDflowline feature of each geospatial point is linked by the unique identification number (*ComID*) of the stream reach. Once the nearest river reach is identified for each reservoir or streamgage, these points are snapped to their nearest NHDflowline. Distance between each snapped point from network headwaters is calculated using the NHDPlusV2 flowlines’ attribute *Arbolate Sum*, which ensures that for all upstream features, the length from headwaters is always increasing from upstream to downstream irrespective of the number of tributaries joining the main path. This distance from headwaters is simply a summation of the *Arbolate Sum* of the nearest NHDflowline feature and the distance of the point (dam or gage) from the end node (upstream node) of this flowline. For faster computation, identification of the nearest flowline is carried out using the R package *sf* ^22^ a C++ ^20^ based *R*^14^ package for simple geospatial analysis. For developing the current dataset, we identify the nearest flowline feature using the following three steps:

1. First, all input shapefiles are transformed to a planar coordinate system Universal Transverse Mercator (UTM). For coordinate system conversion, the UTM zone of each point should be calculated for each watershed region. For simplicity, the zone with the maximum number of points is used for the entire region. This method can lead to erroneous conversion while handling a vast watershed at once, such as Mississippi. We carried out this step separately for each VPU in the NHD*Plus*V2 dataset for CRB.
2. After this, the nearest stream reach is identified for each point, and its *ComID* is noted.
3. Next, each point’s names and the information about the nearest NHDflowline are gathered as text from point shapefiles. These river names are then compared with NHD*Plus*V2 *GNIS_NAME* (Geographic Names Information System) by a robust text comparison method. This attribute is present for the majority of the main stem rivers and their tributaries. This step considers that the point might be located below or upstream or near or at another point or flow reach as well as at a tributary or canal or ditch or dike relative to the main flow path. Whenever no matching string is found at this step based on the nearest flowline identified in step 2, a warning is issued. A warning is also issued whenever NHDP*lus*V2 flowlines *GNIS_NAME* is blank. For points where a warning is issued, additional checks are carried out. First, the distance from these points to all flowlines within a user-specified buffer distance is calculated. Suppose the nearest line feature in step 2 gave a warning. In that case, the algorithm looks for successive nearest line feature, within a buffer distance of 1 km, using a combination of distance comparison and fuzzy string matching^27^ between *GNIS_NAME*s of these flowlines within the buffer distance and the name of a given point (USGS station name for a streamgage; and NID dam name and river names for the reservoirs). This buffer distance is carefully chosen depending on the resolution of the data, units of distance measurement, and the difference between the distances to successive lines from the point of interest. This step issues warning suggesting visual checks which may be manually handled. Visual checks are only issued whenever a new flowline is chosen using fuzzy string matching over the nearest one identified in step 2. To decide on a viable buffer distance and accuracy of the snapping algorithm, we used a subset of national gage locations provided by NHD as a benchmark for linking streamgages with NHDFlowlines. We tested a range of buffer distances 100 m, 200 m, 500 m, 1 km, and 10 km, and counted the number of erroneously assigned flowlines. For the said buffer distances, 5.8–7.5% of streamgages needed correction. The visual warnings for dams are checked manually and corrected if needed. With a 1 km buffer distance, the algorithm issued visual warnings for 45 dams out of 1344. After manually checking each of them, the nearest lines for ten dams needed to be adjusted (see Table SI 1). Overall, if a visual warning is issued for less than 10% of the points, we posit that the snapping process is satisfactory as warnings at this stage do not necessarily mean a wrong flow line is linked to a point. It may merely denote that the name of the NHDflowline feature is missing in NHD*Plus*V2 dataset which is acceptable as not all minor river reaches and small tributaries have an associated river name in NHD*Plus*V2 data. Upon the availability of higher resolution spatial data, this threshold can be made more stringent.

Identification of the nearest NHDflowline feature is critical for the overall reliability of the dataset. Additional validation of this step is carried out by comparing the *ComID* of nearest flow lines of each gage used in the current study, with that of the gages provided in NHD*Plus*V2 dataset. Errors at this step may arise from sources, such as inaccurate coordinate transformation, wrong choice of the buffering distance during point snapping. In this data preparation stage, we join the NHDPlus hydrography datasets for regions 14 and 15 and create a single input data (*NHD_data.RData*).

### Network analysis

Initial network data is created by traversing the *NHDPlusflowlines* network from headwaters to the downstream most node(s) of each watershed, in a parallel fashion (Fig. 2, ‘Network Analysis’ panel). We extensively used the *R*^14^ package dplyr^21^ in this work. We use three NHDPlusv2 database files (.*dbf* files) for each watershed - *NHDflowline*, *PlusflowlineVAA*, and *Plusflow* - using the following steps:

1. In the NHDP*lus*V2 dataset, headwaters or upstream most stream reaches are designated by the attribute *StartFlag* being 1. Headwater flowlines with at least one point are analyzed first (Step 2a in Fig. 2). Points are ordered according to their increasing distance from headwater. The downstream most point of the analyzed headwater flowlines are selected, and from each such point, network analysis is carried out recursively along the downstream direction.
2. At this stage, points downstream of headwater lines but without any upstream points (i.e., reservoirs or streamgages) are also selected for further analysis. Nearest NHDflowlines for each of these points are grouped by *LevelPathID*, ensuring all features along the river segment are uniquely grouped.
3. Next, a parallel and recursive node search along the downstream direction is carried out for each group identified in the previous step using ‘Move downstream’ function (Fig. 2). Input arguments for this function are *x*– points under consideration (node numbers, *ComID* of the nearest flowline, *dP*, *dHW*) and ‘*PATH*’ - ‘main’ or ‘minor’ flow path along which the recursive node search is carried out. For a ‘main’ path (Step 2b in Fig. 2), *LevelPathID* of upstream and downstream NHDFlowline are the same, whereas, for a ‘minor’ path (Step 2c in Fig. 2), *LevelPathID*’s are different for an upstream or downstream stream reach connection. This is essentially a depth-first search along a given river stem and given *PATH* that builds network connectivity from upstream to downstream by recursively updating *x* (Fig. 2). At this iterative step, we account for different conditions such as river junctions (e.g., divergences, convergences, or both), boundary of a watershed, isolated network, and coastlines or end of a stream reach. ‘Move downstream’ function accesses the NHD*PlusV2* VPU tables (*PlusFlow, PlusFlowlineVAA, and NHDFlowlines*).

In this method, the raw or initial output is a connectivity matrix - a *N*_*P*_ × *N*_*P*_ square matrix where *NP* is the total number of points (all dams and gages) analyzed. While the raw output in this format is easy to compute and handle, a connectivity matrix for a large dataset of a riverine network can often be a huge sparse matrix. We present our final dataset as an edge list for better memory usage and ease of incorporating additional information on points and flowlines, which is a popular and efficient representation tool of tree data structure. The edge list consists of nodes and edges information for the entire river network. The nodes are the infrastructures along the flowlines and edges are the connecting river reaches in between successive infrastructure. This highly flexible and robust edge list can be easily used in any traditional network search algorithm. Although we generate a separate edge list for each drainage basin, they can be easily merged by identifying the *ComID*’s of the NHDPlusflowlines that represent the basin outlet(s).

## Data Records

The RICON dataset^17^ for CRB provides (1) a complete list of edges for the unidirectional network connecting streamgages, reservoirs and NHDPlusflowline features for CRB and (2) attributes of all nodes (reservoirs and streamgages) including their geospatial locations, unique identifiers, immediate upstream and downstream nodes, etc. The RICON data archive^17^ contains the dataset tables in comma-separated values (CSV) format.

**Fig. 3:**
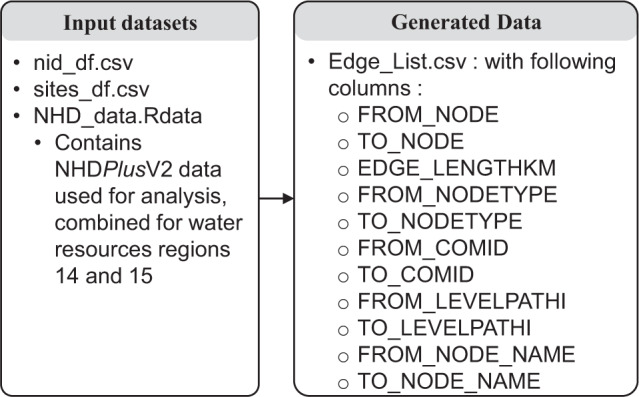
Workflow and relationship between files.

The dataset also contains the NHDPlusV2 dataset used for the analysis (NHD_data). For a quick visualization purpose, the combined spatial data of points (dams and gages linked to Edge_list.csv) are provided along with combined NHDFlowlines data for upper and lower CRB (not shown in Fig. 3).

## Technical Validation

All the codes for this project are written by one person (Sudarshana Mukhopadhyay) in R^14^ language, and the codes are tested in another machine by Chandramauli Awasthi for validation of a successful performance. The codes are also reviewed and documented to guarantee the sequencing along with informative annotations.

The developed RICON dataset^17^ was partially evaluated against selected studies (Table 3) that have identified reservoir-streamgage dependency over CRB^28–30^. Reported relative position between the selected gage stations and dams^28–30^ are in line with the findings in our dataset. This provides a validation of the proposed method and the developed RICON dataset for CRB from this study.

**Table 3:** Snapshot of the output edge list showing relative locations of selected reservoirs and streamgages in CRB.

Name	From Node	To Node	From Node Name	To Node Name	Distance (KM)
Flaming Gorge^29^	Point.09234000	Point.UT10121	Carter Creek at Mouth Near Manila, Utah	UT10121 Flaming Gorge	20.8
	Point.UT10121	Point.09234500	UT10121 Flaming Gorge	Green River Near Greendale, UT	0.62
Gunnison River Near Grand Junction, CO^30^	Point.09144250	Point.09152500	Gunnison River at Delta, CO	Gunnison River Near Grand Junction, CO.	67.6
	Point.09152500	Point.CO83017	Gunnison River Near Grand Junction, CO.	CO83017 Redlands	18.4
San Juan River Near Bluff, UT^29^	Point.09371010	Point.09379500	San Juan River at Four Corners, CO	San Juan River near Bluff, UT	122.3
	Point.09379500	Point.AZ10307	San Juan River near Bluff, UT	AZ10307 Glen Canyon	509.3
Glen Canyon^29^	Point.09335000	Point.AZ10307	Colorado River at Hite, UTAH	AZ10307 Glen Canyon	204.5
	Point.AZ10307	Point.09379910	AZ10307 Glen Canyon	Colorado River below Glen Canyon Dam, AZ	2.02
Colorado River at Lees Ferry, AZ^30^	Point.09379910	Point.09380000	Colorado River below Glen Canyon Dam, AZ	Colorado River at Lees Ferry, AZ	23.7
Hoover^29^	Point.09404200	Point.NV10122	Colorado River Abv Diamond Creek Nr Peach Springs AZ	NV10122 Hoover	203.4
	Point.NV10122	Point.09421500	NV10122 Hoover	Colorado Rv Blw Hoover Dam, AZ-NV	0.16
Davis^29^	Point.09421500	Point.AZ10309	Colorado Rv Blw Hoover Dam, AZ-NV	AZ10309 Davis Bor	107.3
Imperial^29^	Point.09429490	Point.CA10159	Colorado River Above Imperial Dam, AZ-CA	CA10159 Imperial Diversion	0.031
	Point.CA10159	Point.09429500	CA10159 Imperial Diversion	Colorado River Below Imperial Dam, AZ-CA	0.015

Note: Only a few connections and columns are shown.

## Usage Notes

A complete workflow and relation between data files are presented in Figs. 2 and 3, respectively, for proper reproducibility. Figure 3 shows the output information that can be accessed using the edge list. To demonstrate the applicability of the proposed method, we selected the upper and lower CRB as a pilot study area. This data set was originally developed for assessing the cumulative effect of dams in flow alteration in this region^23^. First, we selected 1344 dams from the NID database and 1656 gages. Focusing on intermediate to large dams^15^ with medium and large dams (height >40 feet or storage capacity >1000 Acre-feet) as per ASCE classification^15^, a subnetwork is created such that streamflow sites (1) are located over streams only, not springs or wells; and (2) they must have daily streamflow records (in cubic feet per second)^23^. Considered a subset of the streamflow sites that had at least 15 years of continuous daily streamflow data till 2017 and selected 84 intermediate to large dams represents 83.3% of the total volumetric storage of all dams in upper and lower Colorado, with a median height of 98 feet and median storage capacity of 22350 Acre-feet. Based on the complete edge list, a subnetwork is developed^23^ that has the following information for each reservoir location: (1) immediate upstream dams, (2) immediate downstream dams, (3) streamflow sites between the current dam and its upstream dams, (4) streamflow sites between the current dam and its downstream dams and (5) distance (in kilometers) between each node (reservoir or streamgage).

Using the “EDGE_LENGTHKM” information in the list of edges (“Edge_List.csv”), one can also determine the distance between any pair of points in the network that are hydrologically connected, but not necessarily located immediately upstream or downstream of each other. For example, from Glen Canyon dam (NID ID AZ10307) to Hoover dam (NID ID NV10122), a 592.9 km path is traversed while visiting the ten sites in between and recording length of each edge along the Colorado River. This is achieved by using “find_distance.R” function in the RICON-toolkit^31^. It should be noted that distances along the CRB network are calculated using high-resolution NHD*Plus*V2 dataset, which may result in the overestimation of river reach lengths compared to that calculated using any pre-existing lower-resolution spatial data.

## Supplementary information

Supplementary information

## Data Availability

All the codes for preparing input data and reproducing the edge list file in RICON^17^ are publicly available in RICON-toolkit^31^.
