# Supplementary material for: Developing the hydrological dependency structure between streamgage and reservoir networks
Source: Sci Data. 2020 Oct 1;7:319. doi: 10.1038/s41597-020-00660-6 (PMC7530663; doi:10.1038/s41597-020-00660-6)
Supplement: Supplementary file 1 — Supplementary information [file 41597_2020_660_MOESM1_ESM.pdf]

Supplementary Information for

Developing the hydrological dependency structure between streamgage and reservoir networks

Sudarshana Mukhopadhyay[1], A. Sankarasubramanian[2], and Chandramauli Awasthi[2]

1. Department of Biological and Environmental Engineering, Cornell University, Ithaca, NY, USA.

2. Department of Civil, Construction and Environmental Engineering, North Carolina State University, Raleigh, NC, USA

Corresponding author(s): Sudarshana Mukhopadhyay (sudarshana.besu@gmail.com)

Contents:

1. Table SI 1

Table SI 1 Details of reservoirs in the Colorado River Basin that are manually verified for visual warnings (VFLAG = 2). "IDS" is the unique identifier for a dam same as in "nid_df.csv". "COMID" (and "NEAREST LINE ID") is the COMID of NHDFlowline that is selected using distance comparison and fuzzy string matching. VFLAG is the visual warning flag. "SELECTED COMID" is assigned after manually checking each point for nearest NHDFlowline. "Match" is 1(0) if "COMID" and SELECTED COMID" are identical (or not).

IDS	NID ID	DAM NAME	COMID	VFLAG	NEAREST LINE ID	LONGITUDE	LATITUDE	SELECTED COMID	Match
106	CO01912	ELK LAKE	18279107	2	18279107	-107.459	40.8783	18279107	1
190	CO00787	COVE LAKE	1356696	2	1356696	-107.579	40.2016	1356192	0
194	CO01915	BULL PARK #2	1356434	2	1356434	-107.025	40.114	1356434	1
223	CO00888	SEATON	1356726	2	1356726	-107.184	40.2566	1356726	1
232	UT00284	SOUTH RESERVOIR	3908949	2	3908949	-110.56	39.6149	3908949	1
239	WY00404	BEAVERS	18275645	2	18275645	-107.465	41.1755	18275615	0
274	AZ00057	CLEAR CREEK #1	21746183	2	21746183	-110.643	34.9811	21746183	1
381	CO02189	BAXTER	1337754	2	1337754	-107.674	38.7683	1337994	0
413	UT10126	MIDVIEW (LAKE BOREHAM)	11982377	2	11982377	-110.165	40.1783	11982377	1
416	UT00326	WIGWAM	11975141	2	11975141	-110.008	40.7966	11975141	1
419	WY02038	KILLPECKER DAM CR#1	3386985	2	3386985	-109.2	41.91	3386985	1
429	AZ00142	JOSHUA CROSBY	20657436	2	20657436	-113.244	36.4075	20657436	1
454	WY01282	KEMMERER	3193006	2	3193006	-110.646	41.9422	3193006	1
565	CO01069	WOMMER #1	17034891	2	17034891	-107.589	37.3219	17034891	1
607	CO00574	HOWARD	3230457	2	3230457	-107.956	39.0216	3230457	1
639	WY01461	DETENTION 1392 #4 DAM	18272352	2	18272352	-107.7	41.12	18272364	0
658	AZ00059	MILLET SWALE	21754718	2	21754718	-110.041	34.4306	21754718	1
718	WY01242	GUILD	3267330	2	3267330	-110.668	41.155	3267332	0
726	AZ20010	CROSBY TANK DETENTION DAM	20657220	2	20657220	-113.23	36.45	20657220	1
788	AZ00068	FRYE CREEK RETARDING	21328561	2	21328561	-109.759	32.8288	21328561	1
790	AZ20011	FLAT TOP DETENTION DAM	20655274	2	20655274	-113.13	36.77	20655274	1
805	NM00535	UPPER GILA VALLEY SITE NO. 5 DAM	3076729	2	3076729	-108.55	33	3080219	0
866	AZ10427	CHRISTMAS TREE	20487846	2	20487846	-109.736	33.9101	20487846	1

IDS	NID ID	DAM NAME	COMID	VFLAG	NEAREST LINE ID	LONGITUDE	LATITUDE	SELECTED COMID	Match
895	CA10185	SENATOR WASH	10005026	2	10005026	-114.475	32.89	10005026	1
921	CA10185	SENATOR WASH - NORTH DIKE	10004956	2	10004956	-114.485	32.9163	10004956	1
934	NM00058	SNOW LAKE DAM	2430992	2	2430992	-108.495	33.415	2430992	1
956	UT10150	CEDARVIEW	11976435	2	11976435	-110.132	40.555	11976433	0
958	UT10151	TOWAVE	3521353	2	3521353	-109.748	39.7517	3521353	1
989	UT00385	FERRON DEBRIS BASIN NO. 4	4876845	2	4876845	-111.177	39.0983	4876845	1
1003	AZ20027	SANDS DRAW DETENTION DAM	21306401	2	21306401	-109.38	32.49	21306407	0
1063	UT10111	PETES HOLE	4876501	2	4876501	-111.387	39.293	4876501	1
1099	CO02226	CUSHMAN	18377574	2	18377574	-107.897	37.8883	18377574	1
1106	UT00362	UTAH POWER & LIGHT - HUNTER ASH	4876703	2	4876703	-111.005	39.1633	4876703	1
1122	AZ20028	SLICK ROCK DETENTION DAM	21305967	2	21305967	-109.48	32.72	21303221	0
1144	CO02272	STEAMBOAT SPRINGS WASTEWATER	1354304	2	1354304	-106.911	40.4919	1354304	1
1220	CO02611	FOLLY POND	1357482	2	1357482	-106.884	40.3499	1357562	0
1233	AZ00202	NEW RIVER	20416150	2	20416150	-112.229	33.735	20416150	1
1237	WY01837	RON W. BALL WASTEWATER	18350443	2	18350443	-110.257	42.7802	18350443	1
1265	WY01662	FREMONT LAKE	18331912	2	18331912	-109.834	42.9005	18331912	1
1270	AZ00243	PAGE EFFLUENT PONDS 2A & 2B	3528941	2	3528941	-111.484	36.9127	3528443	0
1274	AZ00225	SLACK	22442418	2	22442418	-111	33.4166	22444076	0
1286	CO02804	WESTERN HILLSIDE RESERVOIR	1319516	2	1319516	-106.542	39.6041	1319516	1
1297	NM00576	GALLUP SPORTS COMPLEX DETENTION DAM	20568773	2	20568773	-108.746	35.5194	20564347	0

IDS	NID ID	DAM NAME	COMID	VFLAG	NEAREST LINE ID	LONGITUDE	LATITUDE	SELECTED COMID	Match
1300	WY02233	KILLDEER WETLAND POND	18315888	2	18315888	-109.467	41.53	18315888	1
1321	NM00634	APS LINED DECANT WATER POND	16967534	2	16967534	-108.512	36.6831	16967546	0
